# Gait dynamics to optimize fall risk assessment in geriatric patients admitted to an outpatient diagnostic clinic

**DOI:** 10.1371/journal.pone.0178615

**Published:** 2017-06-02

**Authors:** Lisette H. J. Kikkert, Maartje H. de Groot, Jos P. van Campen, Jos H. Beijnen, Tibor Hortobágyi, Nicolas Vuillerme, Claudine C. J. Lamoth

**Affiliations:** 1 University of Groningen, University Medical Center Groningen, Center for Human Movement Sciences, Groningen, The Netherlands; 2 Univ. Grenoble Alpes, EA AGEIS, Grenoble, France; 3 MC Slotervaart Hospital, Department of Geriatric Medicine, Amsterdam, The Netherlands; 4 The Hague University of Applied Sciences, Faculty of Health, Nutrition & Sport, The Hague, The Netherlands; 5 MC Slotervaart Hospital, Department of Pharmacy and Pharmacology, Amsterdam, The Netherlands; 6 Utrecht University, Science Faculty, Department of Pharmaceutical Sciences, Utrecht, The Netherlands; 7 Institut Universitaire de France, Paris, France; Semmelweis Egyetem, HUNGARY

## Abstract

Fall prediction in geriatric patients remains challenging because the increased fall risk involves multiple, interrelated factors caused by natural aging and/or pathology. Therefore, we used a multi-factorial statistical approach to model categories of modifiable fall risk factors among geriatric patients to identify fallers with highest sensitivity and specificity with a focus on gait performance. Patients (n = 61, age = 79; 41% fallers) underwent extensive screening in three categories: (1) patient characteristics (e.g., handgrip strength, medication use, osteoporosis-related factors) (2) cognitive function (global cognition, memory, executive function), and (3) gait performance (speed-related and dynamic outcomes assessed by tri-axial trunk accelerometry). Falls were registered prospectively (mean follow-up 8.6 months) and one year retrospectively. Principal Component Analysis (PCA) on 11 gait variables was performed to determine underlying gait properties. Three fall-classification models were then built using Partial Least Squares–Discriminant Analysis (PLS-DA), with separate and combined analyses of the fall risk factors. PCA identified ‘pace’, ‘variability’, and ‘coordination’ as key properties of gait. The best PLS-DA model produced a fall classification accuracy of AUC = 0.93. The specificity of the model using patient characteristics was 60% but reached 80% when cognitive and gait outcomes were added. The inclusion of cognition and gait dynamics in fall classification models reduced misclassification. We therefore recommend assessing geriatric patients’ fall risk using a multi-factorial approach that incorporates patient characteristics, cognition, and gait dynamics.

## Introduction

Approximately 30% of all old adults aged 65 or older experience a fall at least once a year. Falls are associated with pain, functional impairments, morbidity, psychological side effects, and even mortality [1]. Preventing falls therefore remains a health care priority and early identification of individuals at risk is the first step in fall prevention. Older compared with younger adults are more likely to fall due to age-related declines in sensory, cognitive, and neuromuscular function, leading to an impaired gait [2]. Consequently, impaired gait and balance, in addition to demographic characteristics (e.g., gender, age, anthropometry, polypharmacy), are related to falls in community dwelling adults [3].

Age-related slowing of gait is the most documented gait outcome, with habitual gait speed slowing by 16% per decade after age 60 [4–6]. A gait speed below 1.0 m/s signifies potential clinical or sub-clinical impairment, such as mobility impairments, recurrent falling, loss of independence and institutionalization [4]. While most studies are concerned with gait speed as main mobility outcome, gait speed alone may lack sufficient specificity because multiple age- and clinical conditions also induce gait slowing (e.g., low back pain, osteoarthritis, and Parkinson’s disease). In addition to gait speed, a variety of measures can quantify the dynamic nature and time-dependent variations of gait, such as detrended fluctuation analysis [7], sample entropy [8], harmonic ratio [9] and index of harmonicity [10], reflecting the presence of long range correlations, gait predictability, gait symmetry, and gait smoothness, respectively. Each of these gait dynamics reflect a unique characteristic of gait and can be considered as complementary to each other. However, some gait measures are inter-related [11]. For instance, the coefficient of variation of stride time increases as gait speed decreases [12]. Factor analysis takes these inter-relations into account and reduces the dimensionality of the gait data by identifying underlying clusters of gait characteristics. Previous studies identified such gait clusters, reflecting different aspects of gait performance related to speed, variability, rhythm, coordination [13–16]. Hence, extracting properties of gait can provide fundamental insights into the meaning of gait function.

With respect to falling, accuracy of fall prediction models increases when characteristics of gait are included [17,18]. For example, gait smoothness prospectively discriminated fallers from non-fallers in community dwelling old adults with a sensitivity of 68.8% and a specificity of 84.2% [18]. In addition, the accuracy of fall prediction models based on clinical tests commonly used in fall risk assessments such as questionnaires, handgrip strength, and neuropsychological tests, increased by 0.14 when comprehensive gait analysis was added (AUC from 0.68 to 0.82, sensitivity: 70%; specificity: 81% [17]).

The accuracy of fall prediction models may be population-dependent and may not be generalizable to patients admitted to geriatric outpatient clinics. Geriatric patients are referred based on general or specific decline by a general practitioner, and are typically characterized by a combination of physical, psychological, and social problems. Hence, these patients can be considered vulnerable and present with an increased risk for adverse events such as falling, hospitalization, and ultimately death [19]. Geriatric outpatients thus differ from age-matched controls recruited from the community, and multiple comorbidities profoundly affect gait. Geriatric patients do not only walk slower than the clinical threshold of 1.0 m/s [4,20], but chronic conditions also modify gait dynamics. For example, 50% of geriatric patients use polypharmacy, which increases the risk for falls [21,22]. Also, nearly 50% of geriatric patients suffer from osteoporotic vertebral fractures, a condition associated with an increase in thoracic kyphosis, decrease in gait stability, and increased fall risk [23,24]. Moreover, up to 30% of geriatric patients above age 60 present with sarcopenia, which is also associated with gait slowing and an increased fall risk [25]. Finally, the prevalence of cognitive impairment ranges from 22–71% in old adults above age 65 [26] and contributes to slow gait, increased gait variability, decreased gait stability, and increased fall risk [27].

Geriatric patients can thus be characterized by a unique set of variables that increases their risk for a fall. Hence, one approach to identify fallers is by grouping fall risk factors into categories, e.g., demographic characteristics typically assessed in clinical practice, cognitive function, as well as detailed gait performance, and use a multi-factorial data analysis method. Such an approach would allow us to examine the role of each factor in fall risk. Subsequently, it facilitates the development of personalized interventions strategies to modify medication [22], cognition [28], and physical activity levels [6]. The latter interventions can be considered crucial to fall-prone, geriatric patients. The present study therefore aims to statistically model categories of fall risk factors that identify geriatric fallers with the highest sensitivity and specificity, with a focus on gait. To this aim, we pursued two complementary objectives: (1) to identify unique gait properties by extracting underlying clusters from 11 gait measures and remove redundancies in these measures using factor analysis and (2) to examine if the sensitivity and specificity of a fall risk model improves when adding first cognitive measures to demographics, and adding then gait factors identified by the factors analysis. We hypothesized that different gait measures sum into the key features of gait, related to speed and dynamics. Because comorbidities are known to significantly affect geriatric patients’ gait performance, we expect that sensitivity, specificity or both will increase fall classification when gait properties are added to the statistical model.

## Materials and methods

### Study population

The present study included 61 patients (41 women and 20 men) of a database of patients that visited the geriatric dayclinic of the MC Slotervaart Hospital, Amsterdam between 2011 and 2013 [23,29,30]. Patients were admitted to the dayclinic based on a medical referral by a general practitioner and underwent extensive screening for physical, psychological, and cognitive functions. All outcome measures except for gait function, hand grip strength, and fall status were part of standard procedures at the diagnostic geriatric dayclinic of the MC Slotervaart hospital. Inclusion criteria were: age 70 or older. Exclusion criteria were: (1) Inability to walk for at least three minutes without a walking aid, (2) inability to speak and understand the Dutch language, and (3) having mobility disability caused by neurological or orthopedic conditions, limiting function in one or both legs. The Medical Ethical Committee of the MC Slotervaart Hospital approved the study protocol. Written informed consent was obtained from all participants or their legal representatives.

### Outcome measures

#### Determination of fall status

A fall was defined as unintentionally coming to rest on the ground, floor, or other lower level [31]. Patients were interviewed retrospectively about the number of falls over the past year. Also, falls were prospectively registered with a ‘fall calendar’, for which patients were contacted monthly up to 12 monthly by telephone follow-up, with a minimum of 6 months. For patients with an MMSE-score below 24, fall history was obtained from a caregiver. A patient was classified as ‘faller’ when one or more falls occurred retrospectively or prospectively. Because the purpose of the present study was to examine (modifiable) factors involved in fall risk, we aimed to include the whole spectrum of fallers, including retro- and prospective fallers. Therefore, the study design was essentially cross-sectional.

#### Patient characteristics

Demographic information including age, gender, and body mass index (BMI) were recorded. Maximal grip strength of the dominant hand [32], was quantified with a Jamar hand-held dynamometer (average of 3 trials). The number of comorbidities was categorized with the Charlson Comorbidity Index (CCI) [33]. Medications were classified according to the Anatomical Therapeutic Chemical (ACT) codes (WHO, 2013) and quantified as the total number of ‘Fall Risk Increasing Drugs’ (FRIDs), including psychotropic and diuretic drugs [22]. Lateral X-rays of the thoracic spine were analyzed to determine the degree of thoracic kyphosis, indicated by the Cobb angle between the superior endplate of the second thoracic vertebra and the inferior endplate of the twelfth thoracic vertebra [23]. Finally, fall risk was assessed according to the Longitudinal Aging Study Amsterdam (LASA) fall risk profile [34].

#### Cognitive function

Global cognition was assessed with the Mini Mental State Examination (MMSE) with scores below 24 denoting cognitive impairment [35]. The 7-minute screen [36] was administered to assess memory and executive function using the Benton’s Temporal Orientation (BTO), the Enhanced Cued Recall (ECR), the animal verbal fluency and the clock drawing test.

#### Gait performance

All patients walked 160 meters at habitual speed on an 80-meter long hallway. A tri-axial accelerometer (87x45x14 mm; sample frequency 100 Hz; Dynaport® MiniMod, McRoberts BV, The Hague, the Netherlands) was attached to the lower back at the level of the third lumbar spine segment to measure medio-lateral (ML) and anterior-posterior (AP) trunk accelerations. Vertical (V) acceleration signals were not analyzed because peaks in these signals sometimes showed clipping and were therefore unreliable. Acceleration signals were analyzed with custom-made software in MATLAB (version 2014b; The MathWorks, Inc). Except for the calculation of the Sample Entropy, the signals were corrected for horizontal tilt and low-pass filtered with a 2^nd^ order Butterworth filter with a cut-off frequency of 15 Hz. Outliers due to turns were removed from the data using a median filter. We determined 11 gait outcomes, reflecting different and complementary gait properties.

Walking speed was calculated by dividing distance walked by the time. Peak accelerations from AP signals were used to detect time indices of left and right foot contacts. Mean and coefficient of variation (CV) of stride times were computed from the time interval between two consecutive ipsilateral foot contacts. Step consistency was quantified by the standard deviation (SD) of the relative phase between sequential ipsilateral indices of foot contact [37]. Higher SD of the relative phase implies a more inconsistent gait pattern. Long-range correlations between strides were quantified by the scaling exponent α using detrended fluctuation analysis [7]. A value of 0.5 ≥ α ≥ 1 suggests the presence of long-range correlations and signifies that future fluctuations in strides are more accurately predicted by previous fluctuations.

The Root Mean Square (RMS) of the AP and ML acceleration quantified the variability in the magnitude of the trunk accelerations. The Index of Harmonicity (IH) was computed to examine the smoothness (frequency content) of the signal, using spectral analysis. Perfect smooth trunk accelerations would reveal an IH of 1 [10]. To quantify the degree of predictability of trunk acceleration time series, the Sample Entropy (SEn) was calculated [38]. A complete predictable (periodic) signal will adopt a SEn of 0, with a larger SEn representing a less predictable gait.

### Statistical analysis

Principal Component Analysis (PCA) with a Varimax rotation and Kaiser normalization was performed on the 11 gait variables to determine underlying gait properties, and to reduce the dimensionality of the data to unique factors. The number of extracted principal components (PC’s) was determined by analyzing the scree plot which reveals the percentage explained variance by each component (usually referred as ‘factor scores’). PC’s with eigenvalues larger than 1 were considered eligible for inclusion in the final model. The regression coefficients of the extracted PC’s were then used for further analyses [14].

To examine the contribution of different fall risk factors, three Partial Least Squares Discriminant Analyses (PLS-DA) were performed using the PLS_toolbox for MatLab (version 3.7.1; Eigenvector Research Inc.). PLS-DA combines PCA and regression analysis and can handle data consisting of a large number of independent, highly collinear, inter-related variables with relatively few observations (subjects) [39]. Note that such a handling of multicollinearity is important, in particular with respect to gait outcomes (e.g., gait speed and stride time are highly correlated [40]). In the PLS-DA analyses, patient characteristics, cognitive and gait measures represented the independent variables (X), and fall-status the categorical, dependent variable (Y). The analysis seeks to find underlying latent variables (LV’s) to investigate fundamental relations between the matrices X and Y by modelling the covariance structures in these two spaces, and removing common variance. All variables were normalized to unit variance. The optimal number of LV’s was determined using the scree plot and defined at the level where a plateau phase in the goodness of prediction (Q^2^) was reached [39]. Cross-validation was performed using venetian-blind (number of data-splits: 7).

Three models were developed based on: (1) only patient characteristics, (2) patient characteristics and cognitive function, and (3) patient characteristics, cognitive function, and the regression coefficients derived from the factor analysis; the gait factors. Outcome measures of the PLS-DA included scores (individual patients observations) and weights (contribution of fall risk factors to the model), quantifying the relationship between fall risk factors and fall status. The variance explained reflected how variables are clustered within each LV. Classification accuracy of the models was quantified as sensitivity, specificity, and area under the curve (AUC) based on Youden’s criterion, and visualized with receiving operating characteristic (ROC) curves, with an AUC of 1 representing a perfect fit.

## Results

### Patient characteristics

Table 1 shows characteristics for fallers (mean age 80.2±4.7) and non-fallers (78.8±5.1).

**Table 1:** Patient characteristics for fallers and non-fallers (mean ± SD).

Variable	Fallers (n = 25)			Non-fallers (n = 36)			
*Patient characteristics*							
Body Mass Index (kg/m^2^)	27.7	±	4.2	26.0	±	3.5	
Handgrip^a^ (Newton)	23.7	±	8.0	27.2	±	8.8*	
Charlson Comorbidity Index^b^	1.6	±	1.4	1.3	±	1.2	
Longitudinal Aging Study Amsterdam fall risk profile^d^	8.0	±	1.2	2.4	±	0.4*	
Cobb Angle^c^ (degrees)	52.0	±	14.5	50.0	±	12.7	
Fall Risk Increasing Drugs^b^ (number)	1.3	±	1.2	1.3	±	1.4	
*Cognition*							*Scale*
Mini Mental State Examination^a^	23.1	±	4.8	23.8	±	3.7	0–30
Benton’s Temporal Orientation test^b^	19.2	±	6.4	10.0	±	3.3	0–113
Enhanced Cued Recall test^a^	11.7	±	4.1	10.4	±	5.0	0–16
Clock Drawing test^a^	10.1	±	2.5	10.6	±	2.5	0–14
Verbal Fluency test^a^	13.3	±	1.5	14.1	±	0.9	0–40

* p < 0.05.

^a^ A higher score indicates better performance.

^b^ A higher score indicates worse performance.

^c^ Values above >50 affect postural control.

^d^ A score of ≥ 8 points indicates an increased risk for recurrent falling.

### Falls

Retrospective fall data was registered from all 61 patients during the interview. From six patients, follow-up fall calendar data was obtained for less than 6 months, because patients changed address, or withdrawn from participation and did not want to be contacted any longer. The mean follow-up duration was 8.6 months. Twenty-five patients were classified as fallers (41%); 18 retrospective fallers, 19 prospective fallers, and 12 patients fell during the last year as well as during follow-up.

### Gait analysis

Three PC’s with eigenvalues > 1 and absolute factor loadings > 0.4 explained 67.50% of the total variance of the 11 gait measures. PC1 reflected measures related to gait speed, stride times, and the amplitude of trunk accelerations and was labeled ‘pace’. PC2 and PC3 represented measures related to gait variability and coordination respectively, and were labeled ‘variability’ and ‘coordination’ (Table 2). These three identified gait components were then used for the PLS-DA analyses below.

**Table 2:** Loadings of the gait variables (eigenvalue >1 and absolute loadings > 0.4) as revealed by PCA with Varimax rotation.

Gait measures	Pace	Variability	Coordination
Walking Speed	-.848		
Root Mean Square AP	-.844		
Root Mean Square ML	-.820		
Index of Harmonicity ML	.791		
Stride Time	.748		
CV Stride Time	.583	.435	
Step Consistency		.781	
Long range correlations		-.774	
Sample Entropy AP		.677	
Sample Entropy ML			.850
Index of Harmonicity AP			.512

CV = Coefficient of Variation; AP = Anterior-Posterior; ML = Medio-Lateral.

### The PLS-DA models

**Fig 1:**
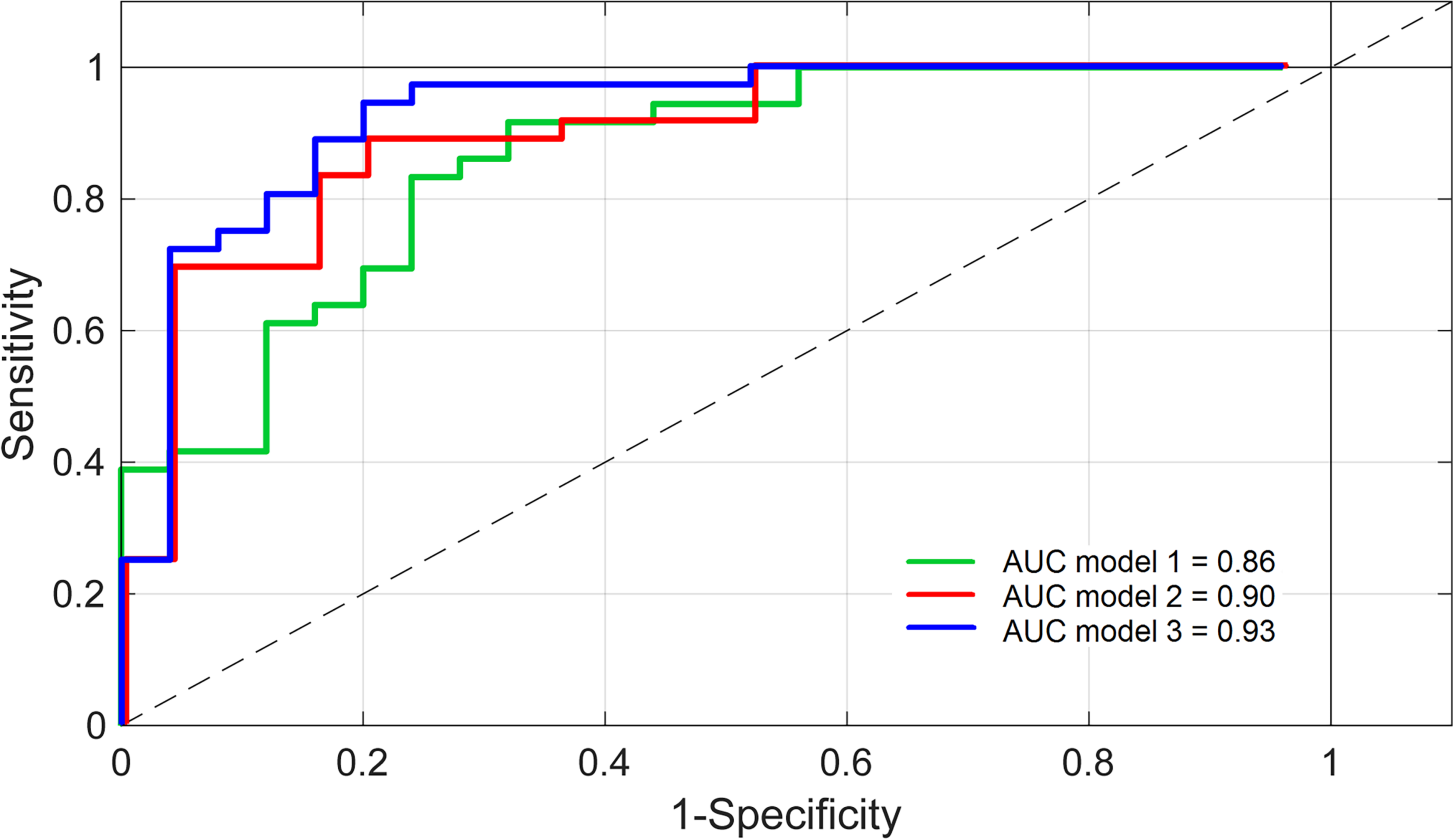
Receiving operating characteristic—Curves for the three fall classification models. Model 1 = Patient characteristics; Model 2 = Patient characteristics + cognitive outcomes; Model 3 = Patient characteristics + cognitive outcomes + gait outcomes. AUC = Area Under the Curve.

Table 3 and Fig 1 show the results of the three PLS-DA models. Model 1 included 3 LV’s, model 2 also included 3 LV’s, and 5 LV’s were extracted for model 3. Note that in all models, LV1 explains most of the variance in the independent variables (X) and falls (Y), followed by LV2 and by LV3, but based on the Q^2^ criteria, five LV’s were included. Classification accuracy of the first model with patient characteristics increased from 0.86 to 0.90 (AUC) when cognitive measures were added. Model accuracy further increased from 0.90 to 0.93 (AUC) when the principal gait components derived were subsequently added. In particular specificity increased in the second model from 60% to 72% and reached 80% when gait measures were included.

**Table 3:** Characteristics of the three PLS-DA models: Number of latent variables, variance explained in X (fall risk factors) and Y (fall-status), and classification accuracy of fallers and non-fallers.

Model	Factors included	Number of LV’s		X-block (%)	Y-block (%)	Sensitivity (%)	Specificity (%)	AUC
1	Patient characteristics	3	LV1	23.7	32.5	92	60	0.86
			LV2	15.0	6.5			
			LV3	15.9	0.7			
			**Sum**	**54.5**	**39.7**			
2	Patient characteristics + cognition	3	LV1	15.1	34.5	89	72	0.90
			LV2	13.1	9.5			
			LV3	20.5	2.1			
			**Sum**	**48.7**	**46.1**			
3	Patient characteristics + cognition + gait	5	LV1	31.8	33.6	92	80	0.93
			LV2	7.8	13.5			
			LV3	18.4	1.3			
			LV4	7.5	1.4			
			LV5	5.3	0.8			
			**Sum**	**52.4**	**50.7**			

LV = Latent Variable; AUC = Area Under the Curve.

Table 4 presents the amount of explained variance per independent variable of each included LV of the final model (model 3). The results signify that X-variables are clustered within the LV’s. Motor performance (gait components and handgrip strength) and the LASA were mainly presented in LV1, cognitive function in LV2 and LV3, and patient characteristics in LV4 and LV5.

**Table 4:** Explained variance (%) per independent variable of the 5 extracted latent variables in model 3.

Independent variable	LV1	LV2	LV3	LV4	LV5	Sum
*Gait*						
Gait Pace	12.2	0.0	6.0	2.2	1.0	22.2
Gait Variability	0.3	5.9	5.5	7.2	2.1	21.0
Gait Coordination	20.4	13.3	1.3	0.4	3.6	39.0
*Cognition*						
Mini Mental State Examination	7.7	0.5	58.7	7.5	4.6	86.6
Benton’s Temporal Orientation test	1.7	0.5	58.7	0.5	1.3	62.7
Enhanced Cued Recall test	6.3	5.5	53.4	0.1	4.8	70.0
Clock Drawing test	9.1	12.9	21.7	0.7	8.5	52.0
Verbal Fluency test	10.4	15.5	28.1	16.1	0.1	70.3
*Patient characteristics*						
Fall Risk Increasing Drugs	2.8	8.2	2.3	18.4	4.5	36.2
Charlson Comorbidity Index	0.2	6.0	9.0	21.1	0.0	36.4
Body Mass Index	3.8	8.3	4.1	28.9	26.4	71.5
Longitudinal Aging Study Amsterdam	74.4	2.4	0.7	1.4	5.6	84.5
Handgrip	40.9	15.6	8.9	0.2	0.6	66.2
Cobb Angle	2.5	0.8	0.0	0.0	10.6	13.9

LV = Latent Variable

**Fig 2:**
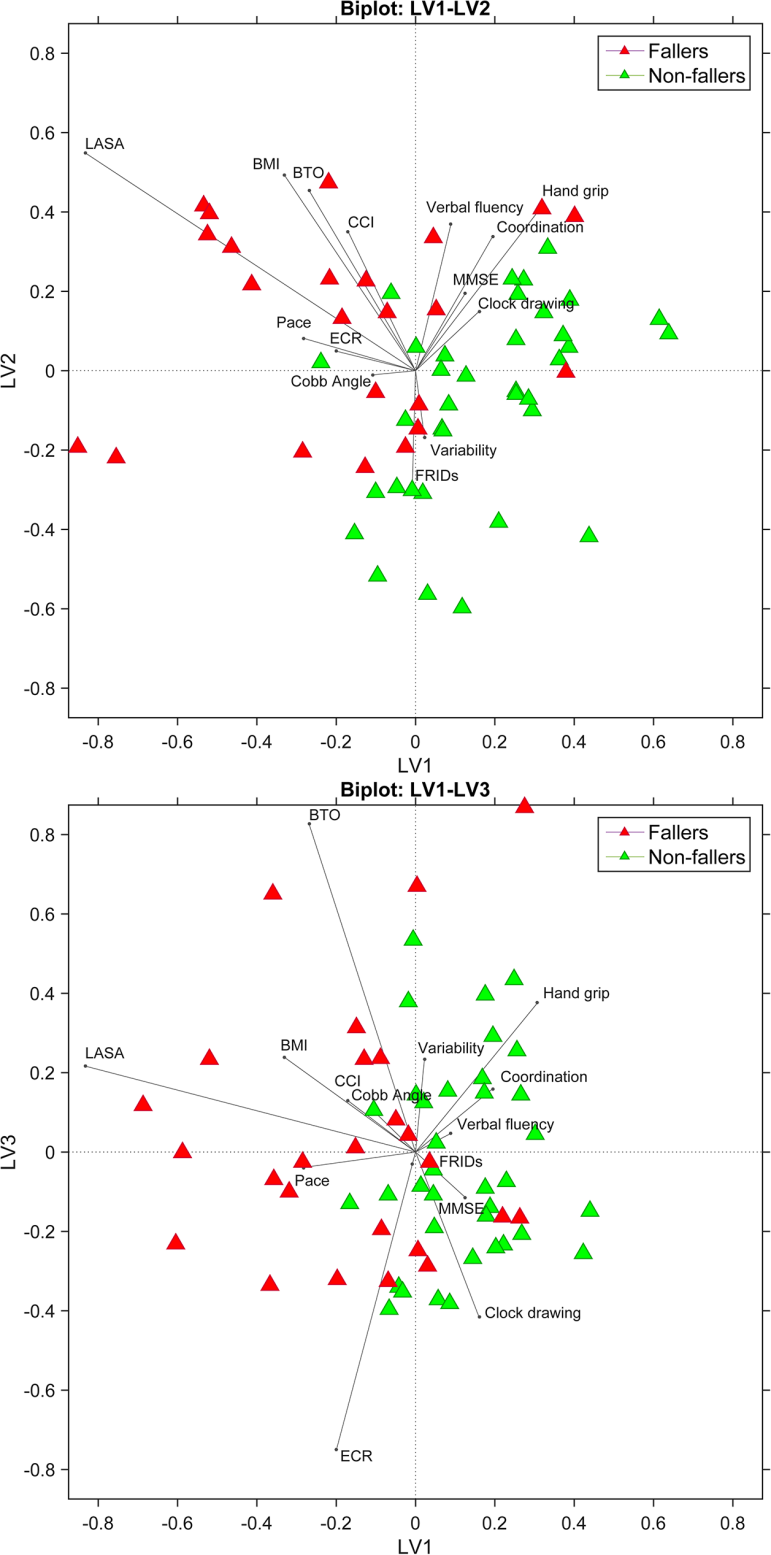
Biplots of latent variables (LV’s) 1 vs. 2 (upper trace) and LV’s 1 vs. 3 (lower trace) provide a graphical representation of the response variable (fall-status) and weights of the independent variables (patient characteristics, cognitive, and gait factors) with respect to the included LV’s. **As clearly shown, fallers and non-fallers (green and red respectively) are clustered. Weight vector size reflects the importance of the variable to the model. The direction of the vector refers to whether variables mainly relate to classification of fallers (sensitivity) or non-fallers (specificity).** BMI = Body Mass Index; CCI = Charlson Comorbidity Index; LASA = Longitudinal Aging Study Amsterdam; FRIDs = Fall Risk Increasing Drugs; MMSE = Mini Mental State Examination; BTO = Benton Temporal Orientation; ECR = Enhanced Cued Recall.

Biplots of the final model provide a graphical representation of the Y-variable (falls) and weights of the X-variables (patient characteristics, cognitive outcomes, and gait outcomes) with respect to the LV’s (Fig 2). Fallers and non-fallers present in sharply separated clusters. The coordinates (size) of the weight vectors reflect the importance of the X-variable to the LV’s. In this figure, the direction of the vectors reflects how these X-variables relate to fallers or non-fallers. The weights show that LASA, BTO, BMI and gait pace are particularly relevant in the identification of fallers, whereas handgrip, clock drawing, verbal fluency, gait variability, and gait coordination are relevant in the identification of non-fallers.

## Discussion

We applied a factor analysis to speed- and dynamic-related measures of gait and we then statistically modeled combinations of factors that classified geriatric fallers with the highest sensitivity and specificity. The factor analysis identified pace, variability, and coordination as key properties of gait. A model that included patient characteristics, cognitive function, as well as gait performance produced high classification accuracy (AUC = 0.93) and showed an increase in specificity from 60% to 80% compared to a model that only included patient characteristics. We discuss how a successful fall risk assessment in the future will most likely include a large array of variables to optimize the identification of fallers among geriatric outpatients.

First, PCA applied to 11 gait variables revealed three unique gait properties: pace, variability, and coordination. ‘Pace’ comprised speed-related measures, namely gait speed, stride time, and the amplitude of AP and ML accelerations (RMS). ‘Variability’ and ‘coordination’ are considered as gait properties that reflect the dynamics of gait and were mainly derived from trunk accelerations. The loading structure was consistent, except for the IH in ML direction, which loaded on the pace component (absolute loading: 0.791) while it was expected to load on the coordination component. This might imply that IH ML is related to gait speed. In general, the extracted components were comparable with components identified by previous studies [13–16].

Second, three PLS-DA models were generated and compared (Table 2 and Fig 1). The first model based on patient characteristics already produced high classification accuracy (AUC = 0.86). LASA clearly outperformed the other variables, as indicated by the size of the weight vectors. LASA provides an extensive screening tool consisting of nine fall-related factors such as dizziness, fear of falling, alcohol intake, fall history, and education level [34]. Although sensitivity of this first model was quite high (80%), specificity remained relatively low (60%). A low specificity (i.e., true negative rate) hampers clinical application because non-fallers will be erroneously identified as fallers and such misclassifications may induce fear of falling and unnecessary interventions.

Adding cognitive measures to the model increased specificity by 12%, to 72% (Table 3). Age-related decline in gait and cognition co-occurs because brain areas that control gait partly overlap with brain areas that control cognitive function [2]. Gait dysfunction can thus be expected in the presence of cognitive impairment [27,41] and an impaired gait control in turn increases fall risk. On the other hand, old adults rely on executive functions in daily activities that require divided attention (e.g., in traffic and walking while talking). Impairment in executive functions may thus cause dangerous situations and increase fall risk.

Adding gait outcomes to the model further increased the models’ specificity by 8%, to 80% (Table 3). Progressive age-related deterioration in neuromuscular and neurophysiological function engenders decline in sensory systems, sarcopenia, slower movement time and central processing, all linked to deficits in gait and balance [42]. In particular gait components ‘variability’ and ‘coordination’ accounted for the increase in specificity, as indicated by the size and direction of the corresponding vectors towards non-fallers (high specificity). These results support the idea that speed-related measures such as gait speed and (CoV) stride time (captured by the pace domain) may be sufficient for classifying fallers only. They do, however, lack specificity that could result in misclassification of non-fallers. Gait speed is widely recognized as an important variable associated with many clinical conditions later in life [4]. The results of the present study show that combining gait speed and speed-related measures with dynamic gait measures will increase specificity and thus classification accuracy. Hence, gait dynamics could be added to measures usually addressed in clinical practice. Nowadays, extensive gait analysis is more easily accessible for clinical practice due to the rapid development of off-the-shelf smartphones, iPods and similar smart devices. Equipped with built-in accelerometers and gyroscopes, the devices are light, inexpensive, easy to handle, and thus suitable to analyze gait in a clinic [43]. However, despite technological advances, we note that future studies should examine the clinical applicability of such smart devices.

While one could question whether the 8% gain in specificity is it worth to add gait performance to the screening assessments, we signify the importance of a correct fall prediction on a clinical level. Misclassification (non-fallers that are classified as fallers) may induce fear of falling and unnecessary interventions and therefore hampers clinical application. Future studies could compare the sensitivity of gait dynamics with existing fall risk tools that examine physical function in the identification of fallers and non-fallers, such as the Physiological Profile Assessment (PPA) test [44]. Such tests may complement each other and reduce the need for excessive testing. In addition, the integration of test results could help to unravel underlying mechanisms of gait dysfunction and neurophysiological changes.

Because of typical challenges associated with clinical research (e.g., recruitment, retention) in this patient group, the sample size of the present study was relatively small (n = 61). As a consequence, because standardization of the follow-up period would induce an in-balance of fallers and non-fallers groups, we choose not to exclude the patients who did not complete the fall calendar for all 12 months. Although exclusion of those patients did not significantly change the results, we recognize this as a potential limitation. Finally, the generalizability of the present study can be considered challenging. However, an increasing number of hospitals is nowadays equipped with a specialized geriatric outpatient clinic. Therefore, assessments used in the present study are often part of regular screening methods, which facilitates applicability and generalizability.

In conclusion, geriatric patients represent a vulnerable population with an increased risk for falling. Fall risk assessment including modifiable fall risk factors revealed high classification accuracy (AUC = 0.93). Although patient characteristics can accurately identify fallers, the evaluation of executive function and gait dynamics reduced misclassification with an increase in specificity from 60% to 80%. Therefore, we underscore the need for a multifactorial approach in fall risk assessment in geriatric patients, including a comprehensive evaluation of patient characteristics, cognitive function, and gait performance. These fall risk factors should ultimately be targeted by individualized interventions to reduce fall risk.

## Supporting information

S1 Dataset The dataset that was used for the analysis, including demographic, cognition, and gait information. (XLSX)
